# Baicalin ameliorates lupus autoimmunity by inhibiting differentiation of Tfh cells and inducing expansion of Tfr cells

**DOI:** 10.1038/s41419-019-1315-9

**Published:** 2019-02-13

**Authors:** Ji Yang, Xue Yang, Jie Yang, Ming Li

**Affiliations:** 1 Department of Dermatology, Zhongshan Hospital, Fudan University, Shanghai, China; 2 Division of Rheumatology, Huashan Hospital, Fudan University, Shanghai, China; 3 Institute of Rheumatology, Immunology and Allergy, Fudan University, Shanghai, China; 4 Blood Engineering Lab, Shanghai Blood Center, Shanghai, China

## Abstract

Baicalin is a natural compound isolated from Chinese herb, which has been reported as an anti-inflammatory drug. Here, we demonstrated that Baicalin treatment could reduce urine protein, inhibit anti-ds-DNA antibody titers, and ameliorate lupus nephritis in MRL/lpr lupus-prone mice. Baicalin inhibited Tfh cell differentiation and IL-21 production, but promoted Foxp3^+^ regulatory T cell differentiation including part of follicular regulatory T (Tfr) cells. Intravenous injection of Baicalin-induced Foxp3^+^ regulatory T cells could relieve nephritis, inhibit Tfh cell differentiation and IL-21 production. Baicalin inhibited mTOR activation, reduced mTOR agonist-mediated Tfh cell expansion and increased Tfr cells. These data suggest that Baicalin attenuates lupus autoimmunity by up- and downregulating the differentiation of Tfr cells and Tfh cells, respectively. Baicalin and ex vivo expanded Foxp3^+^ regulatory T cells are promising therapeutics for the treatment of lupus.

## Introduction

Systemic lupus erythematosus (SLE) is a common autoimmune disease that involves multiple organ systems. The prevalence ranges from 20–150 cases in a population of 100,000 and appears to be increasing because the disease cannot be effectively cured^1^. Drugs such as glucocorticoids and immunosuppressive agents are used to treat SLE, but long-term use can lead to a range of side effects, therefore, it is urgent and necessary to find more safe and effective treatments for SLE.

The autoantibodies formation against nuclear cell components is a typical feature of SLE and therefore fundamental to the pathogenesis of disease. The production of autoantibody relies on T cell-assisted B cell activation. CD4^+^CXCR5^+^PD-1^+^ T follicular helper (Tfh) cells, a CD4^+^ T cell subset mainly locate in germinal centers (GCs), primarily produce IL-21^2–4^. Tfh cells help B cells in GCs become antibody-producing plasma cells or memory B cells, which produce autoantibodies in autoimmune diseases^5–7^. Circulating Tfh cells are increased in the blood of SLE patients and correlate with SLE severity, and increased numbers of Tfh cells lead to increased IL-21 production in lupus-prone mice^8–15^. Thus, inhibition of Tfh cells might reduce autoantibody production during the treat of SLE. CD4^+^CD25^+^Foxp3^+^ regulatory T (Treg) cells are essential for maintaining self-tolerance^16,17^ and play key roles in regulating immune system homeostasis^17^. Forkhead/winged-helix transcription factor Foxp3 is essential for the development and function of CD4^+^CD25^+^ regulatory T cells^18^, induction of the transcription factor Foxp3 can converse CD4^+^CD25^−^ naive T cells to CD4^+^CD25^+^ regulatory T cells^19^. CD4^+^CXCR5^+^Foxp3^+^ follicular regulatory (Tfr) cells are a group of Foxp3^+^ regulatory T (Treg) cells that are located in GCs and share similar phenotypic characteristics with Treg cells and Tfh cells, but work as negative regulators by inhibiting Tfh and B cells^20–23^. Tfr cells function as immunosuppressants and then could be used to reduce inflammation in autoimmune diseases, previous studies indicated that Tfr cells could arise from natural Foxp3^+^Treg cells^21–23^, or from naive T cells^24,25^. Thus, it might be possible to induce Tfr cell expansion in vitro and to use these cells to treat lupus.

Previously, we screened for natural compounds that promoted Foxp3 activity and found that Baicalin, which is extracted from the root of the *Scutellaria* baicalensis Georgi plant (also called Huang Qin in traditional Chinese medicine), could restore Foxp3 expression after IL-6-mediated inhibition and promote Foxp3^+^ Treg cell differentiation^26,27^. Because Tfr cells are derived from Treg cells^21–23^, we speculated that Baicalin might also promote part of Foxp3^+^ Tfr cell differentiation and that these mixed Foxp3^+^ cells might be used to treat lupus. In this study, we examine whether Baicalin treatment can effectively relieve lupus-associated autoimmunity, and the role of Baicalin on differentiation of Tfh and Foxp3^+^ regulatory cells in vitro and in vivo.

## Results

### Baicalin treatment relieves lupus nephritis in MRL/lpr mice

**Fig. 1:**
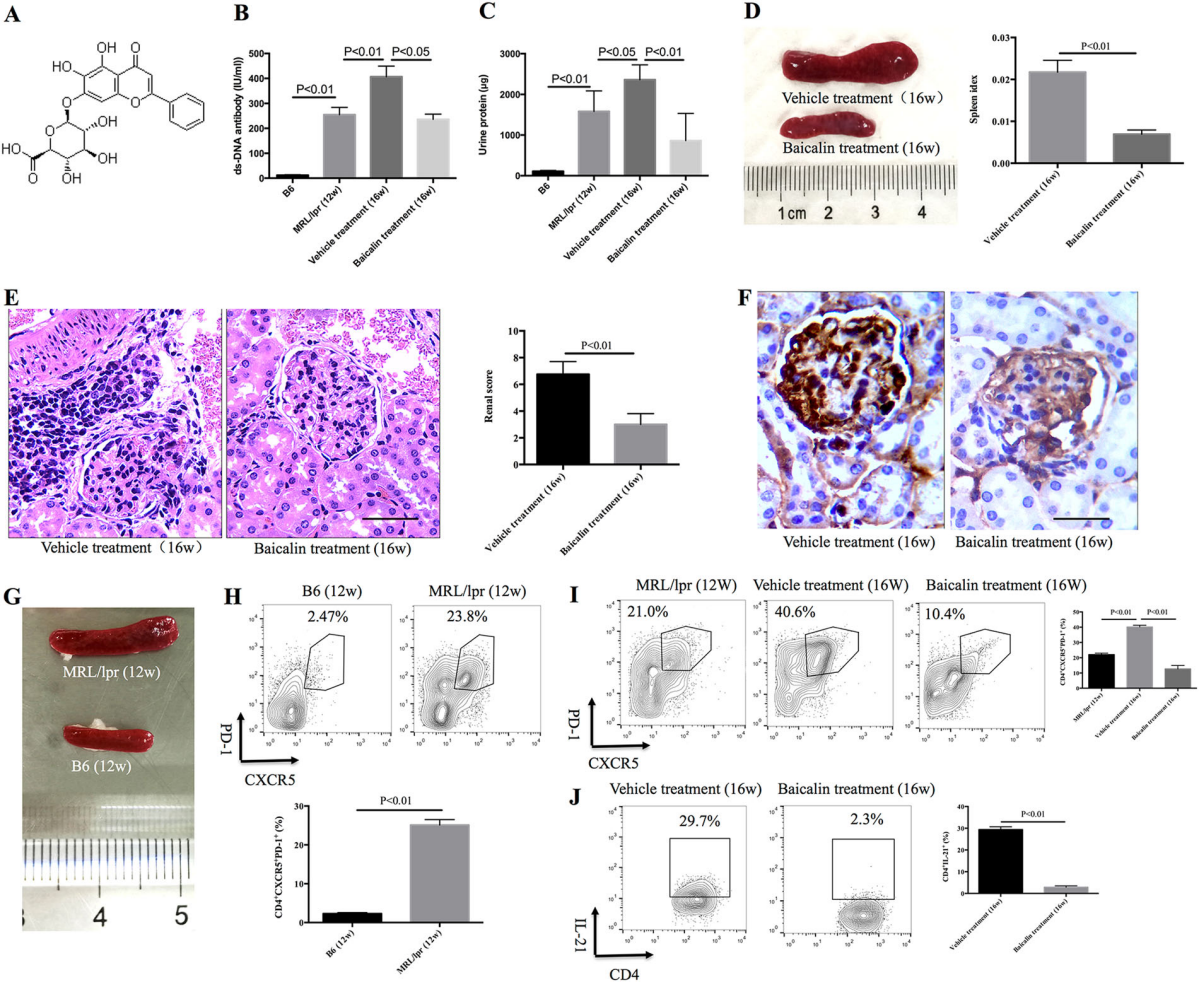
Baicalin treatment relieves lupus autoimmunity and inhibits Tfh cell differentiation in MRL/lpr mice. Twelve-week-old of MRL/lpr mice were treated intraperitoneally with 200 mg/kg Baicalin or PBS vehicle every day for 4 weeks. **a** The chemical structure of Baicalin. **b** Baicalin treatment reduced serum anti-ds-DNA antibody levels (*n* = 4 for each group). **c** Baicalin treatment reduced 24 h protein in urine levels (*n* = 4 for each group). **d** Baicalin treatment inhibited spleen enlargement and reduced the spleen index (*n* = 4 for each group). **e** Baicalin treatment relieved kidney inflammation as visualized by H&E staining (left) and reduced the renal scores (right, *n* = 4 for each group). Scale bar, 100 μm. **f** Baicalin treatment inhibited IgG deposition in kidney. Scale bar, 100 μm. **g** Spleens were enlarged in 12-week-old MRL/lpr mice when compared with age- and sex-matched B6 mice. **h** CD4^+^CXCR5^+^PD-1^+^ Tfh cells were expanded in 12-week-old MRL/lpr mice when compared with B6 mice (*n* = 4 for each group). **i** Baicalin treatment reduced the percentage of CD4^+^CXCR5^+^PD-1^+^ Tfh cells in the spleens of MRL/lpr mice (*n* = 4 for each group). **j** Baicalin treatment reduced the percentage of CD4^+^IL-21^+^ cells in the spleens of MRL/lpr mice (*n* = 4 for each group). 12w and 16w indicate the ages of the mice. ANOVA and Student’s *t*-test were used

Baicalin (7-glucuronic acid, 5, 6-dihydroxyflavone, molecular weight = 446.36. Fig. 1a) is a flavonoid compound originally isolated from the Chinese Herb Huangqin (*Scutellaria* baicalensis Georgi). Twelve-week-old MRL/lpr mice were injected intraperitoneally with 200 mg/kg Baicalin daily for 4 weeks. Baicalin treatment reduced serum ds-DNA titers from an average of 466.1 IU/ml to an average of 236.2 IU/ml and reduced 24 h protein in urine level from an average of 2360.4 μg/24 h to 863.6 μg/24 h (Fig. 1b, c). Baicalin treatment inhibited spleen enlargement and reduced the spleen index (Fig. 1d). Baicalin treatment relieved kidney inflammation, decreased renal scores, and reduced deposition of IgG in the kidney (Fig. 1e, f). These data suggest that Baicalin treatment ameliorated lupus nephritis and reduced the upregulated humoral immune response in vivo.

### Baicalin inhibits Tfh cell differentiation in MRL/lpr mice

Tfh cells aid effector B cells, augment autoimmunity, and are expanded in SLE patients^8–13^, thus inhibition of Tfh cells could be used to treat lupus. In MRL/lpr mice, the spleens were enlarged and the percentage of CD4^+^CXCR5^+^PD-1^+^ Tfh cells was 11.0 times higher in 12-week-old MRL/lpr mice than in sex- and age-matched B6 mice (Fig. 1g, h). During the development of lupus autoimmunity, the percentage of Tfh cells expanded from an average of 22.0% in 12-week-old MRL/lpr mice to 40.2% in 16-week-old MRL/lpr mice (Fig. 1i). The 12-week-old MRL/lpr mice were injected intraperitoneally with 200 mg/kg Baicalin daily for 4 weeks and Baicalin treatment decreased the percentage of Tfh cells to an average of 12.6% (Fig. 1i). Intracellular production of IL-21 in CD4^+^ T cells was blocked by Baicalin treatment (Fig. 1j). These data indicate that Baicalin could inhibit Tfh cell differentiation in MRL/lpr mice.

### Baicalin inhibits Tfh cell differentiation and IL-21 production in vitro

**Fig. 2:**
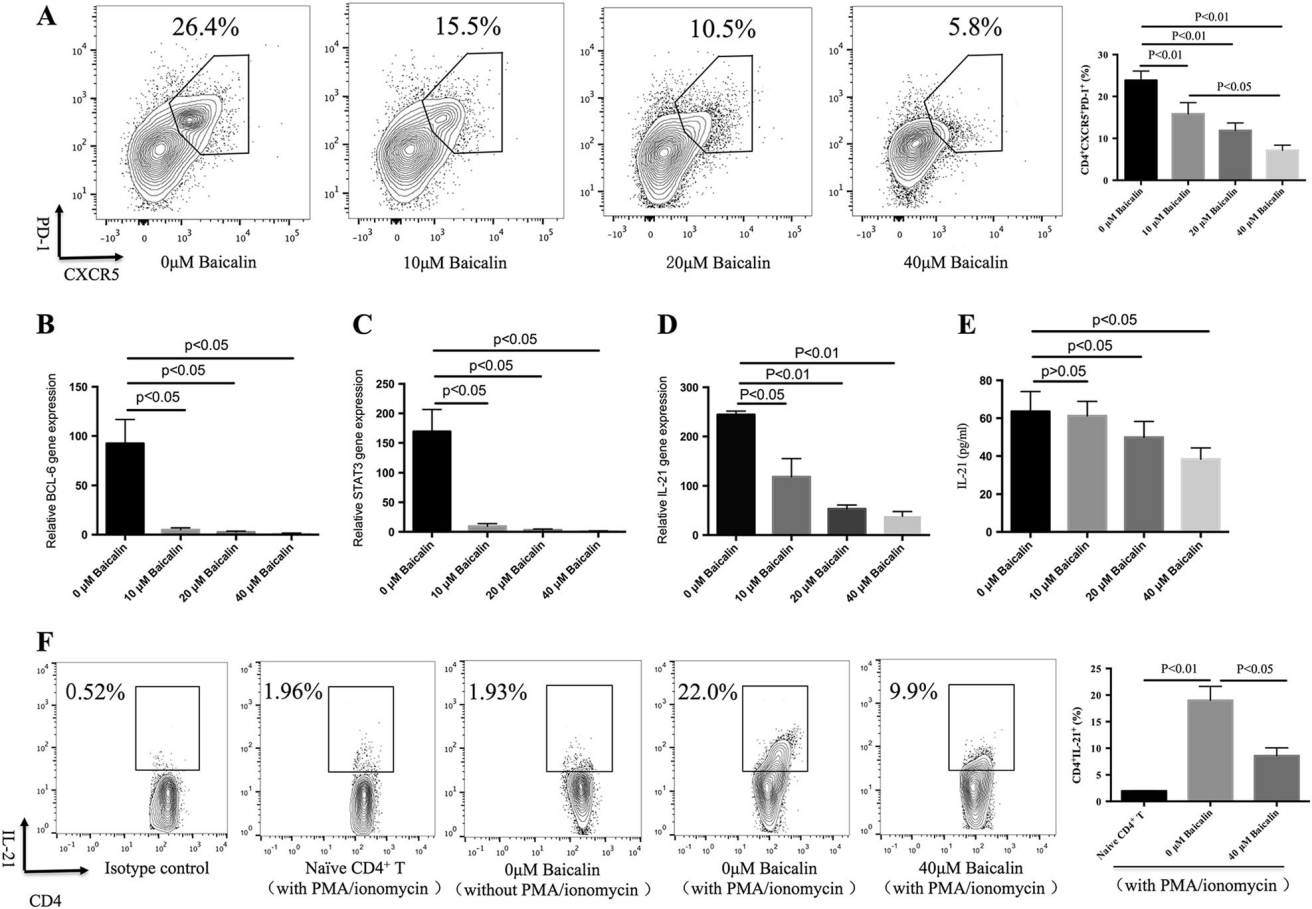
Baicalin inhibits Tfh cell differentiation and IL-21 production in vitro. Naive T cells from MRL/lpr mice were cultured in the presence of IL-21 and IL-6 with or without different doses of Baicalin for 5 days. **a** CXCR5^+^PD-1^+^ cells were analyzed by flow cytometry using a CD4^+^ gate (left). The results of flow cytometry of CD4^+^CXCR5^+^PD-1^+^ cells (right). Results shown are representative of three independent biological experiments. **b** BCL-6, **c** STAT3, and **d** IL-21 gene expressions were analyzed by real-time RT-PCR. Results shown are representative of three independent biological experiments. **e** Naive T cells were cultured in the presence of IL-21 and IL-6 with or without different doses of Baicalin for 5 days. IL-21 production in supernatants was analyzed by ELISA. **f** Naive T cells were cultured in the presence of IL-21 and IL-6 with or without 40 μM Baicalin for 5 days and stimulated with PIB for the last 5 h, intracellular IL-21^+^ expression in CD4^+^ T cells was detected by flow cytometry (left) with the results of the flow cytometry of CD4^+^IL-21^+^ cells (right). Results shown are representative of three biological independent experiments. ANOVA and Student’s *t*-test were used

IL-21 and IL-6 together could induce CD4^+^CXCR^+^PD-1^+^ Tfh cell differentiation, and this Tfh cells could promote immunoglobulin production form B cells (Figure S1), thus Tfh cells could be a therapeutic target for treatment of autoimmune disease. We further investigated the role of Baicalin on Tfh cell differentiation in vitro, the data showed that Baicalin could dose-dependent inhibit Tfh cell differentiation (Fig. 2a). Bcl-6 and STAT3 are important transcription factors for Tfh cell differentiation^2,12,28^, and Baicalin inhibited mRNA expression of both Bcl-6 and STAT3 in cultured Tfh cells in a dose-dependent manner (Fig. 2b, c). Baicalin inhibited IL-21 gene expression and protein secretion during the differentiation of Tfh cells in a dose-dependent manner (Fig. 2d, e). In addition, 40 μM Baicalin could inhibit intracellular IL-21 production in CD4^+^ T cells (Fig. 2f). These data suggest that Baicalin inhibited Tfh cell differentiation and IL-21 production in vitro.

### Baicalin promotes Tfr cell differentiation both in vitro and in vivo

**Fig. 3:**
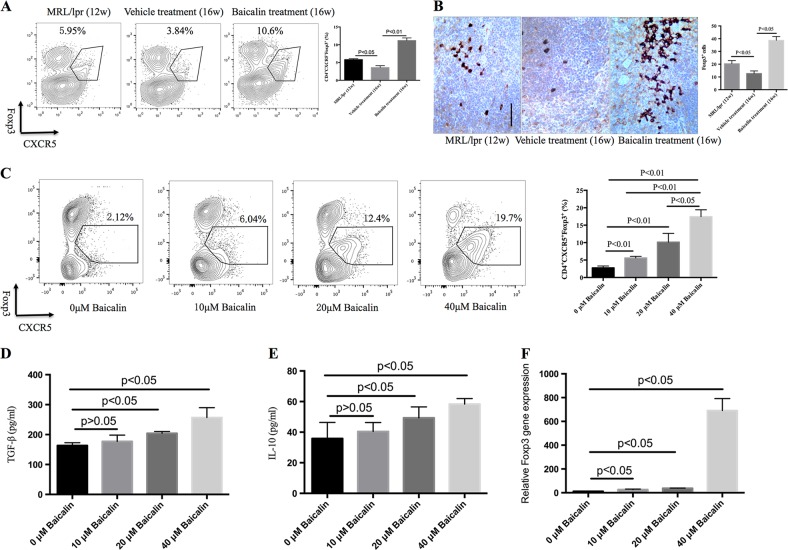
Baicalin promotes CD4^+^CXCR5^+^Foxp3^+^ Tfr cell differentiation. Twelve-week-old of MRL/lpr mice were treated intraperitoneally with 200 mg/kg Baicalin or PBS vehicle daily for 4 weeks. **a** Baicalin treatment increased the percentage of CD4^+^CXCR5^+^Foxp3^+^ cells in the spleens of MRL/lpr mice when compared with vehicle treatment (*n* = 4 for each group). **b** Baicalin treatment promoted Foxp3 protein expression in the spleens of MRL/lpr mice when compared with vehicle treatment (*n* = 4 for each group). Foxp3^+^ cells were counted using ×40 magnification and five independent microscopic fields were selected randomly for each sample to ensure representativeness and homogeneity. Scale bar, 100 μm. **c** Naive T cells from MRL/lpr mice were cultured in the presence of TGF-β and IL-2 with or without different doses of Baicalin for 5 days. The percentage of CD4^+^CXCR5^+^Foxp3^+^ T cells was detected by flow cytometry. Results shown are representative of three biological independent experiments. **d** TGF-β and **e** IL-10 in supernatants was analyzed by ELISA. **f** Foxp3 gene expression was analyzed by real-time RT-PCR. Results shown are representative of three biological independent experiments. 12w and 16w indicate the ages of the mice. ANOVA and Student’s *t*-test were used

Tfr cells are located in GC and function as negative regulators for GC responses by suppressing Tfh and B cells^23^. CD4^+^CXCR5^+^Foxp3^+^ T cells are regarded as Tfr cells in previous studies^29,30^. Here, our data showed that the percentage of CD4^+^CXCR5^+^Foxp3^+^ Tfr cells was lower in 16-week-old MRL/lpr mice than in 12-week-old MRL/lpr mice, but 4 weeks of Baicalin treatment recovered and even increased the percentage of Tfr cells (Fig. 3a). Immunohistochemical staining showed that Baicalin also promoted Foxp3 protein expression in the spleens of MRL/lpr mice (Fig. 3b). These data indicate that Baicalin might exert a therapeutic role in MRL/lpr mice by promoting Tfr cell differentiation in vivo.

A recent study demonstrated that Tfr cells could be induced from naive T cells^24^, our data showed that Baicalin could also induce part of CD4^+^CXCR^+^Foxp3^+^ Tfr cell differentiation in vitro (Fig. 3c). Further data showed that Baicalin-induced Foxp3^+^ regulatory T cells could inhibit the differentiation of CD19^+^CD38^+^ B cell differentiation (Figure S2). TGF-β and IL-10 are two cytokines derived from regulatory lymphocytes that function as immunosuppressants during immune responses^20^, and Baicalin treatment promoted secretion of TGF-β and IL-10 in a dose-dependent manner (Fig. 3d, e). In addition, Baicalin could promote Foxp3 gene expression in vitro (Fig. 3f). These data suggest that Baicalin promoted differentiation of Foxp3^+^ regulatory T cells cells from naive T cells in vitro.

### Baicalin regulates Tfh and Tfr cell differentiation by affecting mTOR activation

**Fig. 4:**
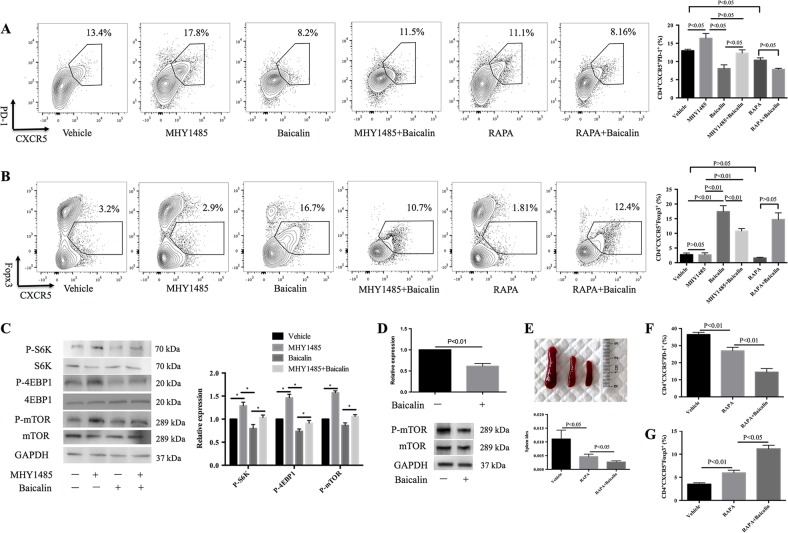
Baicalin inhibited mTOR-mediated Tfh cell expansion. **a** Naive T cells sorted from MRL/lpr mice were cultured in the presence of IL-21 and IL-6 with or without 10 μM mTOR agonist MH1485, 200 ng/ml mTOR antagonist rapamycin (RAPA) or 40 μM Baicalin for 5 days. CXCR5^+^PD-1^+^ cells were analyzed by flow cytometry using the CD4^+^ gate (left). The results of flow cytometry of CD4^+^CXCR5^+^PD-1^+^ cells (right). Results shown are representative of three biological independent experiments. **b** Naive T cells from MRL/lpr mice were cultured in the presence of TGF-β and IL-2 with or without 10 μM MH1485, 200 ng/ml RAPA or 40 μM Baicalin for 5 days. CXCR5^+^Foxp3^+^ cells were analyzed by flow cytometry using the CD4^+^ gate (left). The results of flow cytometry of CD4^+^CXCR5^+^Foxp3^+^ cells (right). Results shown are representative of three biological independent experiments. **c** Sorted naive T cells were cultured with TGF-β, IL-2 with or without 40 μM Baicalin or 10 μM MHY1485 for 3 h, P-mTOR, p-S6K, P-4EBP1, and GAPDH expression were analyzed by western blot. **d** Twelve-week-old of MRL/lpr mice were treated intraperitoneally with 200 mg/kg Baicalin or PBS vehicle daily for 4 weeks. P-mTOR and GAPDH expression in spleen were analyzed by western blot. MRL/lpr mice were treated intraperitoneally with 1.5 mg/kg/d RAPA with or without 200 mg/kg of Baicalin daily for 4 weeks, **e** RAPA and Baicalin treatment inhibited spleen enlargement and reduced the spleen index (*n* = 4 for each group). **f** RAPA and Baicalin treatment reduced the percentage of CD4^+^CXCR5^+^PD-1^+^ Tfh cells in the spleens of MRL/lpr mice (*n* = 4 for each group). **g** RAPA and Baicalin treatment promoted the percentage of CD4^+^CXCR5^+^Foxp3^+^ cells in the spleens of MRL/lpr mice (*n* = 4 for each group). *, *p* < 0.05. ANOVA and Student’s *t*-test were used

Activation of mTOR leads to differentiation of Th17, Th1, and Tfh cells, but inhibits Treg cell differentiation^31–34^. To further analyze the role of the mTOR signaling pathway in Tfh and Foxp3^+^ regulatory T cell differentiation, naive T cells were cultured with or without mTOR agonist MHY1485, mTOR antagonist rapamycin, and with or without Baicalin. MHY1485 promoted Tfh cell differentiation, whereas Baicalin inhibited MHY1485^−^mediated Tfh cell expansion (Fig. 4a). MHY1485 had a tendency to inhibit CD4^+^CXCR^+^Foxp3^+^ T cell differentiation, whereas Baicalin treatment reversed MHY1485-mediated inhibition of CD4^+^CXCR^+^Foxp3^+^ Tfr cells (Fig. 4b). Rapamycin could inhibit Tfh cell differentiation but could not promote Tfr cell differentiation in vitro. In addition, during the differentiation of Foxp3^+^ regulatory T cells, Baicalin treatment could inhibit phosphorylation of mTOR, 4EBP1, and S6K (Fig. 4c). In addition, Baicalin could also inhibit phosphorylation of mTOR in MRL/lpr mice (Fig. 4d). To further verify the role of Baicalin on mTOR in vivo, MRL/lpr mice were treated with Baicalin and mTOR antagonist rapamycin, the data showed that Baicalin and rapamycin synergistic inhibited spleen enlargement, inhibited Tfh cells and promoted Tfr cells than rapamycin used only (Fig. 4e–g). These data suggest that mTOR activation is related to Tfh cell amplification and reduction of Tfr cells and that Baicalin regulates Tfh and Tfr cell differentiation by inhibiting the mTOR signaling pathway.

### Treatment with Baicalin-induced Foxp3^+^ regulatory T cells relieves lupus nephritis

**Fig. 5:**
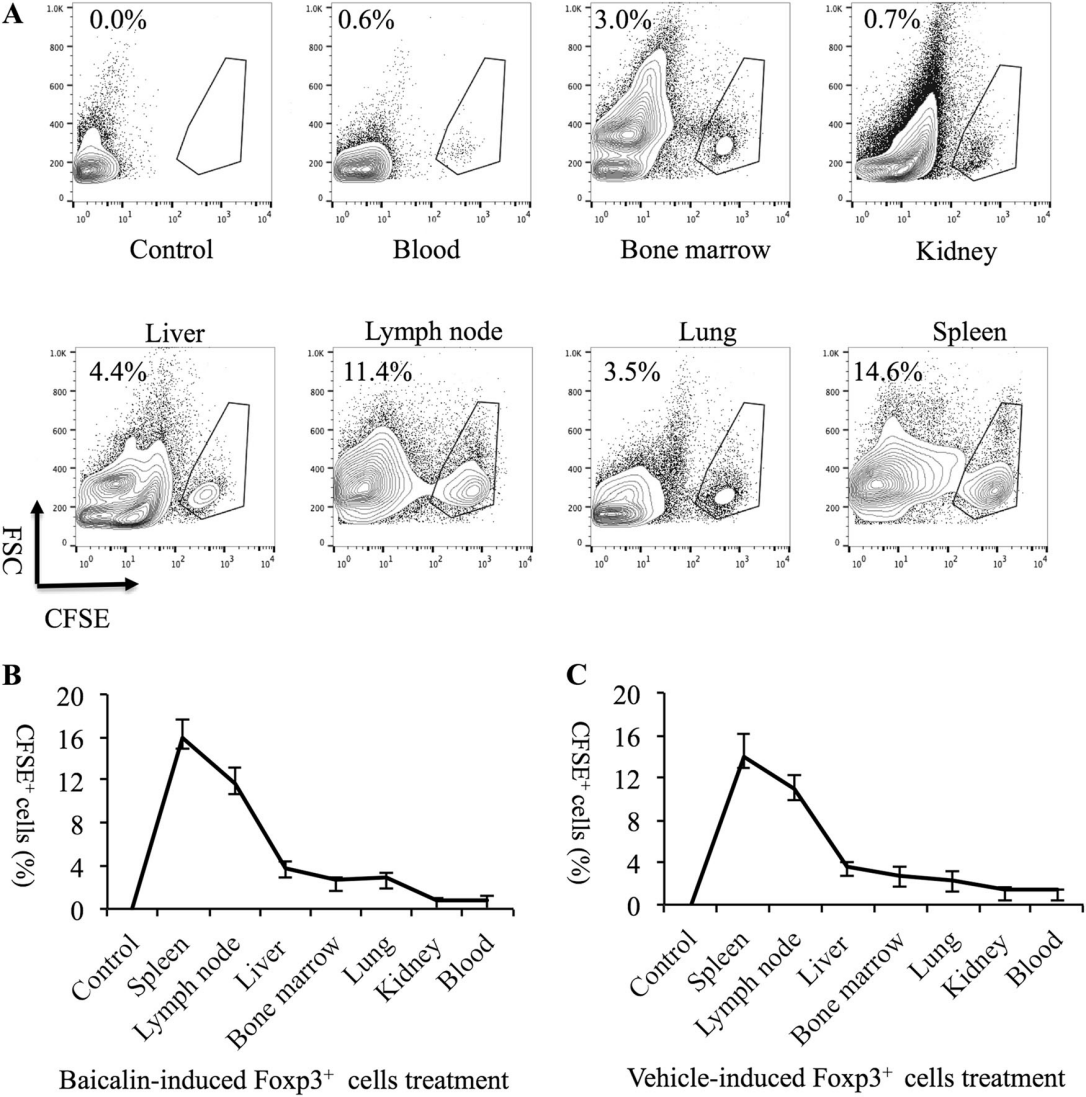
The distribution of intravenously injected Foxp3^+^ T cells in MRL/lpr mice. 1 × 10^6^ Baicalin-induced Foxp3^+^ T cells and 1 × 10^6^ vehicle-induced Foxp3^+^ T cells were labeled with CFSE and injected intravenously into MRL/lpr mice. **a** Three days later, the percentage of CFSE^+^ labeled cells in the spleens, lymph nodes, livers, bone marrow, lungs, kidneys, and blood were analyzed by flow cytometry. **b** The percentage of Baicalin-induced Foxp3^+^ T cells, (**c**) and vehicle-induced Foxp3^+^ T cells in different organs of MRL/lpr mice (*n* = 4 for each group)

1 × 10^6^ Baicalin-induced Foxp3^+^ regulatory T cells or 1 × 10^6^ vehicle-induced Foxp3^+^ regulatory T cells were labeled with CFSE, and then injected intravenously into MRL/lpr mice. Three days later, the mice were sacrificed and the percentage of CFSE^+^ cells in the spleens, lymph nodes, livers, bone marrow, lungs, kidneys, and blood were analyzed by flow cytometry. The data showed that the injected CFSE^+^ cells primarily gathered in the spleens and lymph nodes (Fig. 5a–c).

**Fig. 6:**
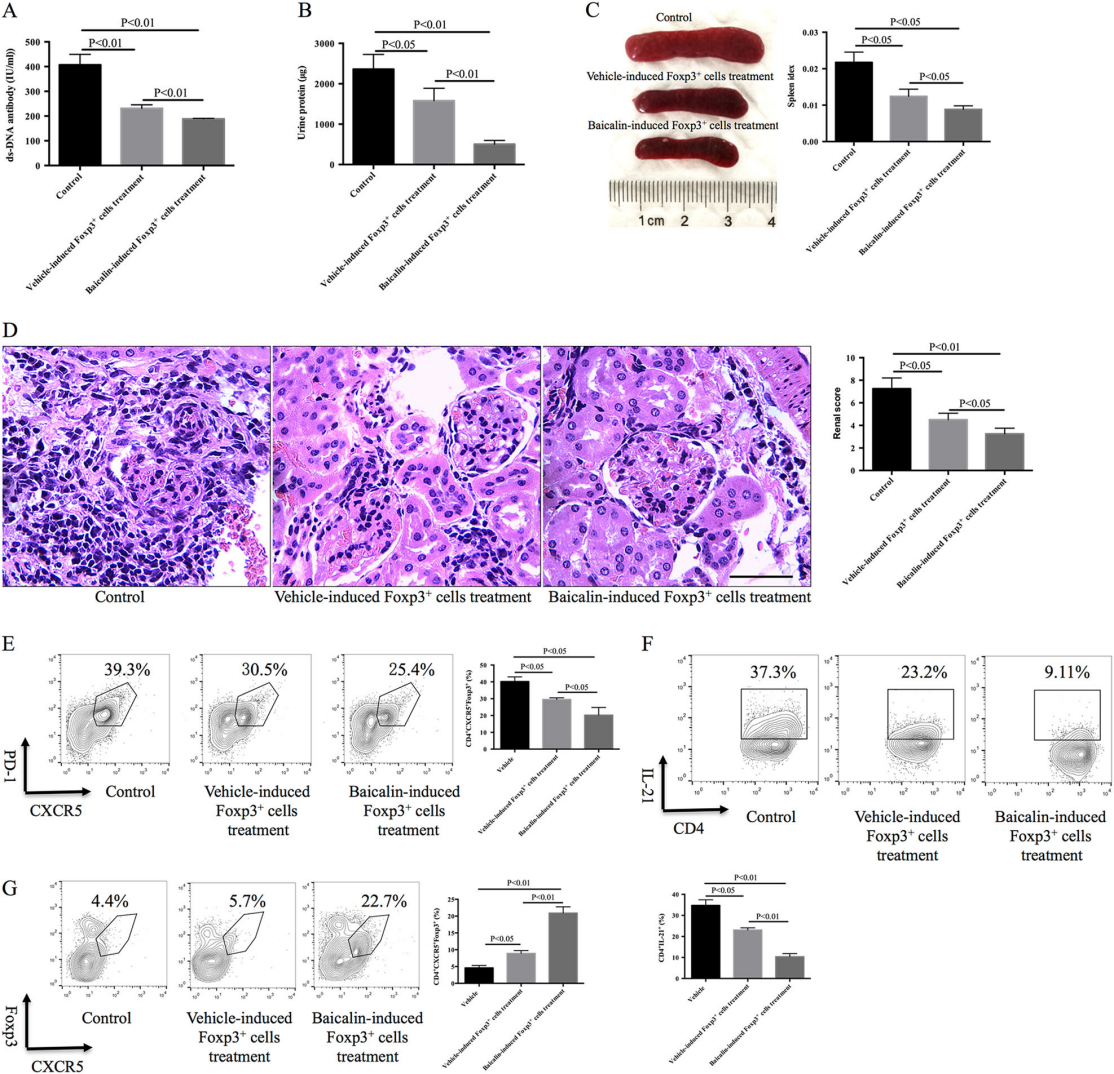
Intravenous injection of *ex vivo* cultured Foxp3^+^ cells relieves lupus autoimmunity. Twelve-week-old MRL/lpr mice were injected intravenously with 1 × 10^6^ Baicalin-induced Foxp3^+^ T cells or 1 × 10^6^ vehicle-induced Foxp3^+^ T cells once a week for 4 weeks. **a** Serum anti-ds-DNA antibody levels were analyzed by ELISA (*n* = 4 for each group). **b** 24 h urine protein levels were detected (*n* = 4 for each group). **c** Spleen enlargement was inhibited and the spleen index was reduced by the treatment of Baicalin-induced Foxp3^+^ T cells (*n* = 4 for each group). **d** Kidney inflammation as analyzed by H&E staining (left) and the renal scores of MRL/lpr mice (right, *n* = 4 for each group). Scale bar, 100 μm. **e** CD4^+^CXCR5^+^PD-1^+^ Tfh cells were analyzed by flow cytometry (*n* = 4 for each group). **f** CD4^+^IL-21^+^ cells were analyzed by flow cytometry (*n* = 4 for each group). **g** CD4^+^CXCR5^+^Foxp3^+^ cells were analyzed by flow cytometry (*n* = 4 for each group). ANOVA and Student’s *t*-test were used

Additionally, after 1 × 10^6^ Baicalin-induced Foxp3^+^ regulatory T cells or 1 × 10^6^ vehicle-induced Foxp3^+^ regulatory T cells were injected intravenously into MRL/lpr mice weekly for 4 weeks, the mice injected with Baicalin-induced Foxp3^+^ regulatory T cells had reduced serum anti-ds-DNA antibody titers, reduced 24 h protein in urine levels, and reduced spleen enlargement when compared with mice injected with vehicle-induced Foxp3^+^ regulatory T cells (Fig. 6a–c). Treatment with Baicalin-induced Foxp3^+^ regulatory T cells for 4 weeks also relieved kidney inflammation and decreased renal scores when compared to the treatment with vehicle-induced Foxp3^+^ regulatory T cells (Fig. 6d). Treatment with Baicalin-induced Foxp3^+^ regulatory T cells reduced the percentage of CD4^+^CXCR5^+^PD-1^+^ Tfh cells, reduced intracellular IL-21 production, and induced CD4^+^CXCR5^+^Foxp3^+^ T cell expansion in vivo (Fig. 6e–g). These data suggest that injection with Baicalin-induced Foxp3^+^ regulatory T cells might be a promising therapeutic method for lupus autoimmunity.

## Materials and methods

### Mice and treatment

Lupus-prone MRL/lpr mice and C57BL/6 (B6) were purchased from Shanghai Laboratory Animal Center (Chinese Academy of Sciences). The mice were fed under pathogen-free conditions. This animal study was approved by the Institutional Animal Care and Use Committee of Zhongshan Hospital, Fudan University. Twelve-week-old MRL/lpr mice were then treated intraperitoneally with 200 mg/kg of Baicalin (purity > 98%, Tauto Biotech, China) dissolved in phosphate-buffered saline (PBS) or with PBS vehicle daily for 4 weeks. For some experiments, MRL/lpr mice were treated intraperitoneally with 1.5 mg/kg/d mTOR antagonist rapamycin (RAPA, LC Laboratories) with or without 200 mg/kg of Baicalin daily for 4 weeks. In addition, 12-week-old MRL/lpr mice were injected intravenously with 1 × 10^6^ Baicalin-induced Foxp3^+^ regulatory T cells or 1 × 10^6^ vehicle-induced Foxp3^+^ regulatory T cells weekly for 4 weeks. Urine was collected for the first 24 h and assayed to detect protein by Coomassie brilliant blue according to the manufacturer’s instructions (Nanjing Jiancheng, China). Four weeks after treatment, the MRL/lpr mice were sacrificed and the spleen index spleens were calculated [spleen index = spleen weight (g)/body weight (g)]. The percentages of CD4^+^CXCR5^+^PD-1^+^ Tfh cells and CD4^+^CXCR5^+^Foxp3^+^ Tfr cells in the spleens were analyzed by flow cytometry. Spleen and kidney tissues were fixed for histopathological assessment.

### Naive CD4^+^ T-cell isolation and Tfh and Foxp3^+^ regulatory T cell differentiation

Naive CD4^+^T cells were isolated from spleens of MRL/lpr mice by using the Dynabeads Untouched Mouse CD4 Cell Kit (Life Technologies AS, Norway). For Tfh cell differentiation, naive CD4^+^T cells were cultured for 5 days with 10 ng/ml IL-21 (PeproTech, USA), 10 ng/ml IL-6 (PeproTech), and 25 µl of anti-CD3/anti-CD28 antibody (BD Biosciences Pharmingen, USA) with various doses of Baicalin (dissolved in DMSO and diluted with PBS) or a DMSO-PBS vehicle as a control. For Foxp3^+^ regulatory T cell differentiation, sorted naive CD4^+^T cells were cultured for 5 days with 5 ng/ml TGF-β (PeproTech), 30 U/ml IL-2 (PeproTech, USA), and 50 ng/ml anti-CD3/anti-CD28 antibody (BD Biosciences Pharmingen) with or without Baicalin as described above. For some differentiation experiments, 10 μM mammalian target of rapamycin (mTOR) agonist (MHY1485; MedChem Express, USA),or 200 ng/ml mTOR antagonist rapamycin (LC Laboratories) was added with 40 μM Baicalin for 5 days.

For some experiment, sorted naive CD4^+^T cells were cultured for 5 days with 5 ng/ml TGF-β, 30 U/ml IL-2, and 50 ng/ml anti-CD3/anti-CD28 antibody with or without 40 μM Baicalin for 5 days, then these 1 × 10^6^ Baicalin-induced Foxp3^+^ regulatory T cells or 1 × 10^6^ vehicle-induced Foxp3^+^ regulatory T cells were injected intravenously into MRL/lpr mice weekly for 4 weeks.

### Flow cytometry analysis

To detect Tfh cells, cells collected from mice spleens or in vitro cultures were stained with APC-conjugated anti-CXCR5, FITC-conjugated anti-CD4, and PE-conjugated anti-PD-1 (all from BD Biosciences Pharmingen) for 30 min and then CXCR5^+^PD-1^+^ cells were detected using flow cytometry with a CD4^+^ gate. To analyze intracellular IL-21 expression, cells were cultured for 5 h with 750 ng/ml ionomycin (Sigma-Aldrich) and 50 ng/ml phorbol myristate acetate (PMA; Sigma-Aldrich, USA) in the presence of 20 μg/ml brefeldin A (Sigma-Aldrich), resuspended in a fixation/permeabilization solution and stained intracellularly with PE-conjugated anti-IL-21, according to the manufacturer’s instructions (eBioscience, CA).

To detect CD4^+^CXCR5^+^Foxp3^+^ cells, cells collected from mice spleens or in vitro cultures were stained with FITC-conjugated anti-CD4 and APC-conjugated anti-CXCR5 (all from BD Biosciences Pharmingen) for 30 min and analyzed using a CD4^+^ gate. Intracellular Foxp3 expression was measured using the PE-Foxp3-staining kit (eBioscience). In some experiments, 1 × 10^6^ Baicalin-induced Foxp3^+^ regulatory T cells or 1 × 10^6^ vehicle-induced Foxp3^+^ regulatory T cells were stained with carboxyfluorescein succinimidyl ester (CFSE) and intravenously injected into MRL/lpr mice. Three days later, the mice were sacrificed and the percentage of CFSE-labeled cells in the spleen, lymph node, liver, bone marrow, lung, kidney, and blood were analyzed by flow cytometry.

### Cytokine detection

Sorted naive T cells were cultured under Tfh or Foxp3^+^ regulatory T cell differentiation conditions with or without different doses of Baicalin for 5 days. IL-21, TGF-β, and IL-10 concentrations were determined by ELISA (eBioscience). Serum ds-DNA antibody level in MRL/lpr mice was detected by ELISA (eBioscience).

### Histopathological assessment

The spleens and kidneys of the mice were fixed, embedded, and stained with hematoxylin and eosin (H&E). The kidneys were stained with IgG (Cell Signaling Technology, Beverly, MA).

The H&E-stained kidney slides were read and interpreted in a blinded fashion as our previous study^26^. Detailed pathological assessments were performed as described previously^35^.

### Western blot

Sorted naive CD4^+^T cells were cultured for 3 h with 5 ng/ml TGF-β, 30 U/ml IL-2, and 50 ng/ml anti-CD3/anti-CD28 antibody with or without 40 μM Baicalin or 10 μM MHY1485. Cells were lysed, and proteins were extracted and blotted with antibodies to p-mTOR (Ser2448), mTOR, p-4EBP1, 4EBP1, p-S6K, S6K, and GAPDH (all from Cell Signaling Technology, Beverly, MA). The protein expressions of p-mTOR (Ser2448), mTOR in spleens of MRL/lpr with or without Baicalin were also analyzed. The proteins were detected with SuperSignal West Pico Chemiluminescent Substrate solution (Thermo Scientific, Rockford, IL).

### RNA isolation and real-time RT-PCR

Total RNA was purified using the RNA Prep Pure Cell Kit according to the manufacturer’s instructions (Tiangen Biotech, China). Then, cDNAs were synthesized using the Fast Quant cDNA RT Kit and mRNA expression was measured using the SYBR Green Quant qRT-PCR Kit (all from Tiangen Biotech, China). The 2^−ΔΔCt^ method was used to normalize transcription to β-actin and to calculate the fold-induction relative to the control. The primer pairs were used: Mus β-actin, forward GAGACCTTCAACACCCCAGC, reverse ATGTCACGCACGATTTCCC; Mus Bcl-6, forward CCTGAGGGAAGGCAATATCA, reverse CGGCTGTTCAGGAACTCTTC; Mus STAT3, forward CCGTCTGGAAAACTGGATAACT, reverse CCCTTGTAGGACACTTTCTGCT; and Mus Foxp3, forward CCCAGGAAAGACAGCAACCTT, reverse TTCCACAACAAGGCCACTTG.

### Statistical analysis

Quantitative data were expressed as mean ± standard deviation. Differences determined by two-tailed Student’s *t*-test were used for two-group comparisons. ANOVA followed by Bonferroni post-hoc test was used for multiple comparisons. All *p* values < 0.05 were considered significant.

## Discussion

Baicalin possesses anti-inflammation, anti-allergy, and hepatoprotective properties and contributes to the treatment of inflammatory diseases, including allergic diseases, hepatitis, and arthritis^36–38^. Baicalin also functions as a potent antioxidant that protects tissues from oxidative stress damage caused by H_2_O_2_, Ca^2+^ flux, hypoxia, high glucose, or ultraviolet light^39,40^. In this study, we showed that treatment with Baicalin for 4 weeks reduced 24-h urine protein levels, relieved kidney inflammation, and inhibited serum anti-ds-DNA antibody production and kidney IgG deposition in MRL/lpr mice. These data indicate that Baicalin is an effective and promising drug for the treatment of lupus autoimmunity, which is consistent with our previous findings^26^.

Our previous studies indicated that Baicalin inhibited the differentiation of Th17 cells and promoted the differentiation of Treg cells^26,27^. Tfh and Tfr cells are two subsets of lymphocytes with reciprocal regulatory functions^9,20^. Until now, the effects and mechanisms of Baicalin on these reciprocal functions in lupus remained unknown. Tfh cells function in B-cell activation and antibody production, and because it has been shown that Tfh cells are expanded in SLE patients and lupus-prone mice^8,10,12–14^, inhibition of Tfh cell differentiation could relieve lupus autoimmunity^41–43^. In this study, we demonstrated that Baicalin inhibits Tfh cell differentiation both in vitro and in vivo. IL-21, a key cytokine produced by Tfh cells, promotes reactions in GCs that skews Tfr to Tfh cells^9^. In this study, we demonstrated that Baicalin treatment inhibits IL-21 production both in vitro and in vivo.

Tfr cells are a subset of CXCR5-expressing Treg cells located in GCs that suppress Tfh cell differentiation, B-cell activation, and antibody production^21–23^. Tfr cells play important regulatory roles in the pathogenesis of autoimmune diseases, such as SLE, rheumatoid arthritis, and graft-versus-host disease^11,23^. Tfr cells express high levels of Foxp3, Bcl-6, CXCR5, PD-1, and ICOS^23^. Although differentiation of Tfr cells is not clearly understood, several studies have shown that Tfr cells could be derived from nTreg cells^21,22^. Recent studies have shown that Tfr cells could arise from naive T cells^24^, and that TGF-β and IL-2 are critical for Tfr cell differentiation^44^. In this study, we showed that co-stimulatory signals, anti-CD28 and anti-CD3 antibodies, together with TGF-β and IL-2 induced CD4^+^Foxp3^+^ regulatory T cell differentiation and that supplementation with Baicalin enlarged the number of differentiated CD4^+^Foxp3^+^ regulatory T cells. Interestingly, Baicalin together with TGF-β, IL-2 and anti-CD3/CD28 stimulation could also induce parts of CD4^+^CXCR5^+^Foxp3^+^ Tfr cell expansion, and this regulatory T cells could inhibit B cell differentiation. The percentage of Tfr cells was lower in 16-week-old MRL/lpr mice than in 12-week-old MRl/lpr mice indicating that the continued lupus autoimmunity correlated with a reduction in Tfr cells. Interestingly, Baicalin treatment effectively recovered the percentage of CD4^+^CXCR5^+^Foxp3^+^ Tfr cells in MRL/lpr mice. Our previous data demonstrated that Baicalin induces Foxp3 expression from naive T cells and promotes Treg cell differentiation in vitro and in vivo^26,27^. Here, our data further indicated that Baicalin could help to induce part of naive T cells to differentiate into Foxp3^+^Tfr cells.

Oxidative stress leads to an imbalance in the immune system that plays an important role in SLE pathogenesis^31,45^. Activation of mTOR has been detected in lupus T cells^33^, and mTOR is a critical integrator of environmental stimuli, which regulates the T-cell signals that influences their differentiation and activation^31,33^. mTOR drives the proinflammatory expansion of Th1, Th17, and Tfh cells and inhibits the development of CD4^+^CD25^+^Foxp3^+^ Treg cells^31,34^. Previous study has showed that Baicalin could inhibit the activation of mTOR in caner cells^46^. Here, our data also showed that Baicalin could also inhibit phosphorylation of mTOR, 4EBP1, and S6K during regulatory T cell differentiation. In addition, mTOR agonist MHY1485 could enlarge the number of differentiated Tfh cells and inhibited Foxp3^+^ regulatory T cell differentiation in vitro, and Baicalin treatment could inhibit mTOR agonist-mediated Tfh cell expansion and recover Foxp3^+^ regulatory T cell differentiation. Reversely, mTOR antagonist rapamycin could inhibit Tfh cell differentiation, and Baiclain in synergy with rapamycin could increase inhibitory effect on Tfh cell differentiation both in vitro and in vivo of MRL/lpr mice. These data suggest that the Baicalin restores the balance of Tfh and Foxp3^+^ regulatory T cells by inhibiting the mTOR signaling pathway. Rapamycin did not promote Tfr cell differentiation as expected in vitro studies, one possible reason is that rapamycin partially inhibited the expression of CXCR5, but not Foxp3 in vitro. However, rapamycin treatment could promote Tfr cells in vivo. We speculated that the increased Tfr cells might attribute to improvement of disease activity and alleviated inflammatory microenvironment caused by rapamycin treatment. These results further indicated that the immune microenvironment in vivo is more complex, and the differentiation of immune cells is often affected by specific immune microenvironment and the interaction of cytokines.

We also showed that Baicalin promoted Foxp3^+^ regulatory T cell differentiation accompanied by inducing TGF-β and IL-10 production, these cytokines might further help Foxp3^+^ regulatory T cells to exert immunosuppressive effects. *Ex vivo* expanded Foxp3^+^ regulatory T cells were injected intravenously into lupus mice, and the injected Foxp3^+^ regulatory T cells could be detected in the spleens, lymph nodes, livers, bone marrow, lungs, kidneys, and blood of the MRL/lpr mice, but mainly migrated into spleens and lymph nodes. Thus, these Foxp3^+^ regulatory T cells could further modulate the immune system and relieve the autoimmunity in vivo. These data suggest that Baicalin-induced Foxp3^+^ regulatory T cells could exert immunosuppressive effects to relieve lupus autoimmunity. We believe that the therapeutic effect might be attributed to mixed Foxp3^+^ T cells including Treg (CXCR5^−^ Treg) cells and Tfr (CXCR5^+^ Treg) cells, further studies should be done to make clear the functional differences of these different Foxp3^+^ cells.

In summary, our data indicated that treatment with Baicalin could relieve lupus autoimmunity by inhibiting Tfh cell differentiation and promoting Tfr cell differentiation. Baicalin could become a promising therapeutic medicine for the treatment of SLE because Baicalin stimulated expansion of Foxp3^+^ regulatory T cells and ex vivo expanded Foxp3^+^ regulatory T cells could be used to treat lupus.

### Ethics statement

The animal study was approved by the Institutional Animal Care and Use Committee of Zhongshan Hospital, Fudan University.

## Supplementary information

Supplemental Figure legend

Figure S1

Figure S2
