# Supplementary material for: Baicalin ameliorates lupus autoimmunity by inhibiting differentiation of Tfh cells and inducing expansion of Tfr cells
Source: Cell Death Dis. 2019 Feb 13;10(2):140. doi: 10.1038/s41419-019-1315-9 (PMC6374440; doi:10.1038/s41419-019-1315-9)
Supplement: Supplementary file 1 — Supplemental Figure legend [file 41419_2019_1315_MOESM1_ESM.docx]

**Supplemental Figure legend**

**Figure S1. Tfh cells promote immunoglobulins production from B cells.** B cells were isolated from spleens of B6 mice by using CD19 Microbeads, and then co-cultured at a 1:1 ration with induced Tfh cells (IL-21+IL-6 for 5 days) in the presence of anti-IgM and anti-CD40 for 3 days. The concentrations of IgM and IgG1 were analyzed by ELISA. Results shown are representative of three biological independent experiments. Student’s *t*-test was used.

**Figure S2. Baicalin-induced Foxp3^+^ T cells inhibit CD19^+^CD38^+^ B cell differentiation.** B cells were isolated from spleens of B6 mice by using CD19 Microbeads, and co-cultured at a 1:1 ration with Baicalin-induced Foxp3^+^ T cells (TGF-β+IL-2+Baicalin for 5 days) in the presence of anti-IgM and anti-CD40 for 3 days. Then, the percentage of BV421-conjugated anti-CD38 among PE-conjugated anti-CD19 was analyzed by flow cytometery (left). The results of flow cytometry of CD19^+^CD38^+^ cells (right). Results shown are representative of three biological independent experiments. Student’s *t*-test was used.
