# Supplementary figures and images for: Baicalin ameliorates lupus autoimmunity by inhibiting differentiation of Tfh cells and inducing expansion of Tfr cells
Source: Cell Death Dis. 2019 Feb 13;10(2):140. doi: 10.1038/s41419-019-1315-9 (PMC6374440; doi:10.1038/s41419-019-1315-9)

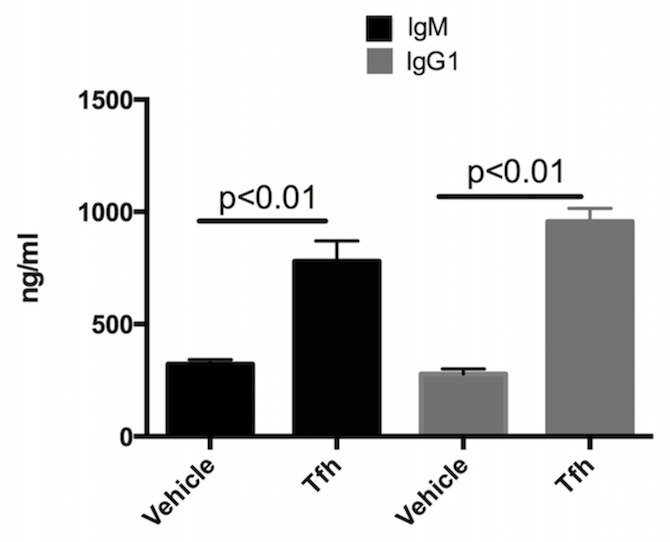

Supplement: Supplementary file 2 — Figure S1 [file 41419_2019_1315_MOESM2_ESM.tif]

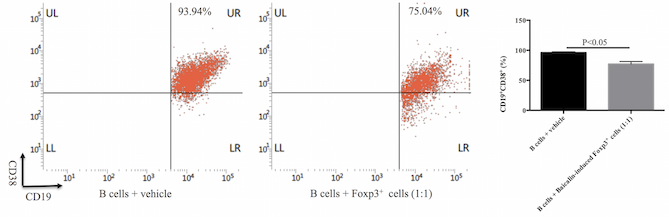

Supplement: Supplementary file 3 — Figure S2 [file 41419_2019_1315_MOESM3_ESM.tif]
